# Christmas with the Wounded and Sick

**Published:** 1916-01-01

**Authors:** 


### CHRISTMAS WITH THE WOUNDED AND SICK.

No one, from the highest to the lowest, will question the fact that all without exception in these days desire to take their part in cheering our warriors at the Front, and in providing all possible care, skilful treatment, and pleasant surroundings for the wounded who have returned to the Motherland to recover, God willing, from their injuries or maladies. In ordinary times, with increasing conviction, animation, and success, Christmas-time has been made memorable throughout our voluntary hospitals by the devoted personal service of an ever-increasing number of the best amongst us. These workers with readiness and conviction have devoted the whole of their time on Christmas Day, and many of them on succeeding days, to an attempt to cheer the sick, and help them to forget their sufferings by worthily celebrating the greatest of Christian festivals. This year the number of hospitals and the buildings converted for use as hospitals have multiplied. In consequence the call for personal service has been so extended as to make it wonderful that everywhere throughout the country the lot of the patients in our hospitals on Christmas Day 1915 has been made blessed, and will be remembered by them as one of the brightest and happiest days of their lives.

We have often insisted that the ideal way to spend Christmas is to devote the day to personal service in some hospital. Even for the elders, a visit to one of our great hospitals at this time should impress them with the spirit they may find within hospital walls. Nor can such visitors fail, in exact proportion to their powers of observation, to receive inspiration and become animated by the feeling that under these conditions Christmas Day can be made memorable by its lessons and exhilarating by the blessedness which such experiences should yield. Throughout the London hospitals, and in the majority, if not in all, the military hospitals and kindred institutions, splendid efforts were made to brighten the Christmas of the wounded and to cheer the sick, both warriors and civilians. In most hospitals there was special Christmas fare for those who were in a condition to partake of it, whilst the patients who wished to were granted the privilege not only of seeing their friends but of smoking in the wards. In some the Christmas Day entertainments were confined to the children's wards, where there were Christmas-trees, decked with presents, brilliantly lighted, presenting charming effects to the eye. This was the case at the London Hospital and in some of the children's hospitals.

